# Clinical characteristics and mortality risk among critically ill patients with COVID-19 owing to the B.1.617.2 (Delta) variant in Vietnam: A retrospective observational study

**DOI:** 10.1371/journal.pone.0279713

**Published:** 2023-01-20

**Authors:** Thanh Van Do, Toshie Manabe, Giap Van Vu, Vuong Minh Nong, Yuji Fujikura, Dung Phan, Thach The Pham, Cuong Duy Do, Tra Thu Doan, Nguyen Trung Nguyen, Thai Quoc Nguyen, Thanh Van Dong, Chinh Quoc Luong, Hiroki Manabe, Dan Kambayashi, Anh Viet Hoang, Nhan Van Vu, Giang Kim Trinh, Son Ngoc Do, Takeshi Kamiya, Hirotaka Ohara, Chi Van Nguyen, Tuan Quoc Dang, Koichiro Kudo, Co Xuan Dao

**Affiliations:** 1 Center for Tropical Diseases, Bach Mai Hospital, Hanoi, Vietnam; 2 Nagoya City University Graduate School of Medicine, Nagoya city, Aichi, Japan; 3 Nagoya City University West Medical Center, Nagoya city, Aichi, Japan; 4 Hanoi Medical University, Hanoi, Vietnam; 5 Respiratory Center, Bach Mai Hospital, Hanoi, Vietnam; 6 Department of Internal Medicine, National Defense Medical College, Tokorozawa, Saitama, Japan; 7 Department of Medical Risk Management and Infection Control, National Defense Medical College Hospital, Tokorozawa, Saitama, Japan; 8 Faculty of Pharmacy and Pharmaceutical Sciences, Monash University, Parkville, Victoria, Australia; 9 Intensive Care Center, Bach Mai Hospital, Hanoi, Vietnam; 10 Poison Control Center, Bach Mai Hospital, Hanoi, Vietnam; 11 Outpatient Department, Bach Mai Hospital, Hanoi, Vietnam; 12 Center for Emergency Medicine, Bach Mai Hospital, Hanoi, Vietnam; 13 Department of Emergency and Critical Care Medicine, Hanoi Medical University, Hanoi, Vietnam; 14 Faculty of Medicine, University of Medicine and Pharmacy, Vietnam National University, Hanoi, Vietnam; 15 Shitennoji University, Habikino city, Osaka, Japan; 16 Showa Pharmaceutical University, Machidashi, Tokyo, Japan; 17 Training and Direction of Healthcare Activities Center, Bach Mai Hospital, Hanoi, Vietnam; 18 Center for Critical Care Medicine, Bach Mai Hospital, Hanoi, Vietnam; 19 Yurin Hospital, Tokyo, Japan; 20 Waseda University, Organization for Regional-Interregional Studies, Tokyo, Japan; Clinica Luganese Moncucco, SWITZERLAND

## Abstract

**Background:**

SARS-CoV-2 Delta variant caused a large number of COVID-19 cases in many countries, including Vietnam. Understanding mortality risk factors is crucial for the clinical management of severe COVID-19.

**Methods:**

We conducted a retrospective study at an intensive care center in Ho Chi Minh City that urgently built by Bach Mai Hospital during the COVID-19 outbreak in Vietnam, when the Delta variant predominated. Participants were laboratory-confirmed patients with SARS-CoV-2 infection, admitted in August 2021. Data on patients’ demographic and clinical characteristics, radiographic and laboratory findings, treatment, and clinical time course were compared between survivors and non-survivors. Risk factors to mortality were assessed using logistic regression.

**Results:**

Among 504 eligible COVID-19 patients, case fatality was 52.2%. Unvaccinated patients accounted for 61.2% of non-survivors and 43.6% of survivors (*p* < 0.001). The time from onset to hospital admission was 8 days in non-survivors and 7 days in survivors (*p* = 0.004). Among non-survivors, 90.2% developed acute respiratory distress syndrome (ARDS). Oxygen therapy was administered for all patients, but antiviral agent was given to 51.7% of non-survivors. 54.2% of non-survivors tested positive for the bacterial infection using blood culture. The risk factors for mortality were diabetes mellitus, respiration rate, oxygen saturation, vaccination status, time from onset to admission, and older age.

**Conclusions:**

Critical patients with COVID-19 owing to the Delta variant in Vietnam had delayed hospital admission, leading to ARDS and death. Early availability of vaccines and preventing bacterial infections are crucial for reducing mortality of COVID-19, especially in low- and middle-income countries.

## Introduction

At the end of 2019, the novel severe acute respiratory syndrome coronavirus 2 (SARS-CoV-2) emerged, and its spread has resulted in a global pandemic, with more than 618 million confirmed cases of coronavirus disease 2019 (COVID-19) and over 6.5 million COVID-19-related deaths as of 9 October 2022 [1]. In Vietnam, the number of COVID-19 cases was relatively small, with no deaths, during the first 7 months of the pandemic. In contrast, over 4.3 million and 2.5 million cases, respectively, were reported in the United States and Brazil, with a case fatality rate of approximately 3.5% in both countries [2, 3]. However, following an epidemic in summer 2021, the second year of the pandemic, the cumulative number of COVID-19 cases in Vietnam increased rapidly, reaching over 961,000 cases by the 1^st^ November, 2021, particularly owing to the emergence of the B.1.617.2 (Delta) variant of SARS-CoV-2 [4, 5].

The SARS CoV-2 Delta variant is estimated to be approximately 60.0% more transmissible than the SARS-CoV-2 Alpha variant [6]. Delta variant is also associated with increased disease severity and higher viral load [7, 8]. This may be attributable to a combination of the high transmissibility of the Delta variant, fatigues on people caused by the prolonged period of pandemic, and an insufficient vaccine supply [8]. However, similarly to other RNA viruses, SARS-CoV-2 continuously mutates, with new variants emerging with ongoing transmission [5, 9, 10]. Therefore, understanding the clinical presentations and risks of disease severity of COVID-19 owing to the SARS-CoV-2 Delta variant is urgently required to improve clinical preparedness for future epidemic waves of COVID-19 as well as future mutant virus strains that are associated with increased transmissibility and disease severity.

The aims of the present study were to elucidate the clinical presentations and mortality risk factors of COVID-19 during the epidemic of the SARS-CoV-2 Delta variant in Vietnam among critically ill patients with COVID-19.

## Methods

### Study design and participants

We conducted a retrospective observational study in an intensive care unit (ICU) center of the national Bach Mai Hospital (BMH) in Ho Chi Minh City, Vietnam. The BMH is originally located in Hanoi and is a tertiary hospital designated as the central (Level 1) hospital in northern Vietnam by the Ministry of Health of Vietnam [11]. This site was established following to the dramatic rise in the number of patients with COVID-19 and severe condition of Ho Chi Minh City in July 2020. Following the dramatic rise in the number of patients with COVID-19 in Ho Chi Minh City in July 2020, the Ministry of Health (MOH), Vietnam issued a decision to establish a COVID-19 Intensive Care Unit (ICU) Center on July 30, 2021 under BMH, named Field Hospital Number 16. The BMH ICU center had 500 beds at the peak of the epidemic and subsequently reduced the number of beds to 360. The ICU center closed on October 15, 2022, after a decrease in the number of COVID-19 cases. The Director of BMH was also the Director of the ICU center. Similarly, the Deputy Director of the Emergency Department and the Deputy Director of the ICU Department of BMH became the Deputy Directors of the BMH ICU Center. The BMH sent over 570 of their staff members, including 240 doctors from the ICU, emergency, infectious, respiratory, nutrition, artificial kidney, neurology, anti-infection, hematology, biochemistry, and microbiology departments.

Study participants were patients who were admitted in the BMH ICU center in Ho Chi Minh City during a month of August 2020. All patients tested positive for SARS-CoV-2 infection using real-time reverse transcriptase polymerase chain reaction and required to treat using mechanical ventilator. Owing to the healthcare system rules in Vietnam [11], BMH, which is a Level 1 hospital, can only receive patients transferred from lower level hospitals, such as provincial and district hospitals. Patients with COVID-19 in the community cannot directly access the BMH ICU center without first presenting to another medical facility [11]. Therefore, our study only included patients who had received medical treatment prior to arriving at the BMH ICU center.

We collected demographic and baseline clinical patient data, including symptoms and signs, laboratory and radiographic findings, vaccination status at the time of admission, clinical time course, complications, drug and oxygen therapies, and clinical outcomes. The primary outcome of the study was in-hospital mortality and the secondary outcomes were risk factors to mortality.

### Statistical analysis

Data are reported as number and percentage for categorical variables and as median with interquartile range (IQR: 25%–75%), or mean with standard deviation (SD) for continuous variables. We compared the data of survivors and non-survivors using the chi-square test or Fisher’s exact test for categorical variables and Mann–Whitney *U* test, one-way analysis of variance, Student *t*-test, Wilcoxon signed-rank test, or Kruskal–Wallis test for continuous variables, as appropriate. Survival curves for the duration of hospitalisation and time from onset to hospital discharge among survivors and non-survivors were analysed using the Kaplan–Meier method; comparisons were made using the log-rank test.

Risk factors associated with mortality were estimated using a logistic regression model that included independent variables of general patient characteristics as well as baseline variables at the time of admission with a *p* value < 0.05 in univariate analysis for survivors and non-survivors. A step-wise selection method was used to select variables using the forced entry method.

Data were analyzed using IBM SPSS version 27 (IBM Corp., Armonk, NY, USA). For all analyses, significance levels were two-tailed, and *p* < 0.05 was considered statistically significant.

#### Ethics approval and consent to participate

The present study was approved by the Ethical Committee which is an Institutional Review Board (IRB) of BMH. Written informed consent was waived by the Ethical Committee for this retrospective chart review, with public notification of the study made using posters.

#### Inclusivity in global research

Additional information regarding the ethical, cultural, and scientific considerations specific to inclusivity in global research is included in the (S1 Checklist).

## Results

### Demographic and clinical characteristics in non-survivors and survivors of COVID-19

During the observation period, a total of 504 patients with COVID-19 (42.1% male patients) were admitted to the study site and were eligible for inclusion in the present study. Among them, 263 patients died during hospitalisation; the case fatality rate was 52.2%. We compared the demographic and clinical characteristics of non-survivors and survivors of COVID-19, as shown in Table 1.

**Table 1:** Demographic and clinical characteristics of patients with COVID-19.

	Non-survivors (n = 263)	Survivors (n = 241)	*p* value
**Demographic**			
Gender—Male, n (%)	113 (43.0)	99 (41.0)	0.668
Age–median (IQR)	64 (57–73)	55(45–64)	< 0.001
<20	0 (0.0)	8 (3.3)	< 0.001
20–39	14 (5.3)	36 (14.9)	
40–59	78 (29.7)	111 (46.1)	
60–79	134 (51.0)	71 (29.5)	
≥80	37 (14.1)	15 (6.2)	
Smoking, n (%)	31 (11.8)	24 (10.0)	0.511
Alcohol. n (%)	12 (4.6)	14 (5.8)	0.527
Pregnant, n (%)	3 (1.1)	0 (0.0)	0.096
BMI—median, IQR)	24.8 (22.1–26.9)	23.9 (21.5–26.3)	0.034
**Vaccination status**			< 0.001
Zero doses	161 (61.2)	105 (43.6)	
1 dose	98 (37.3)	119 (49.4)	
2 doses	4 (1.5)	17 (7.1)	
**Comorbidities**			
No. of Comorbidities			< 0.001
0	67 (25.5)	111 (46.1)	
1	71 (27.0)	59 (24.5)	
2	86 (32.7)	48 (19.9)	
≥3	39 (14.8)	23 (9.5)	
Stroke	20 (7.6)	5 (2.1)	0.004
Dementia	0 (0.0)	4 (1.7)	0.052
Diabetes mellitus	84 (31.9)	42 (17.4)	< 0.001
Hypertension	151 (57.4)	103 (42.7)	< 0.001
Cardiovascular disease	58 (22.1)	41 (17.0)	0.155
COPD	18 (6.8)	12 (5.0)	0.377
Asthma	2 (0.8)	1 (0.4)	1.000
High cholesterol	6 (2.3)	5 (2.1)	0.874
Liver failure	12 (4.6)	8 (3.3)	0.475
Renal failure	7 (2.7)	5 (2.1)	0.666
Malignant	4 (1.5)	0 (0.0)	0.055
HIV	1 (0.4)	0 (0.0)	1.000
Tuberculosis	5 (1.9)	3 (1.2)	0.726
Pregnant	3 (1.1)	0 (0.0)	0.250
**Rout of transmission**			0.003
Direct contact from family members	69 (26.2)	69 (28.6)	
Direct contact from other than family	28 (10.6)	37 (15.4)	
Co-workers	4 (1.5)	16 (6.6)	
unknown	162 (61.6)	119 (49.4)	

Abbreviation: IQR, interquartile range; BMI, body mass index; COPD, chronic obstructive pulmonary disease.

The median age of survivors was younger than that of non-survivors (*p* < 0.001), with 65.1% of non-survivors aged ≥ 60 years. Among the total patients, 266 (52.8%) were not vaccinated; 61.2% of non-survivors were unvaccinated. The prevalence of patients who were partially or fully vaccinated was higher among survivors than non-survivors (*p* < 0.001). Only 1.5% of non-survivors and 7.1% of survivors were fully vaccinated. In total, 64.1% of survivors had no comorbidities whereas 47.5% of non-survivors had two and more comorbidities (*p* < 0.001). In total, 61.6% of non-survivors and 49.4% of survivors did not know the cause of infection (*p* = 0.003).

### Clinical and laboratory findings at hospital admission among non-survivors and survivors of COVID-19

The results of a comparison of clinical presentations and laboratory findings at the time of hospital admission among non-survivors and survivors of COVID-19 are shown in Table 2.

**Table 2:** Clinical presentation and laboratory test results at the time of hospital admission among patients with COVID-19.

	Non-survivors (n = 263)	Survivors (n = 241)	*p* value
**Symptoms at admission**			
Fever, mean (SD)	36. 9 (0.476)	36.7 (0.422)	< 0.001
Fever (≥38.0℃)	16 (6.1)	7 (2.9)	0.088
Dry Cough	182 (69.2)	159 (66.0)	0.439
Productive cough	137 (52.1)	106 (44.0)	0.069
Sore throat	85 (32.3)	73 (30.3)	0.624
Sneeze	43 (16.3)	33 (13.7)	0.405
Dyspnea	181 (68.8)	145 (60.2)	0.042
Olfactory	79 (30.0)	89 (36.9)	0.101
Gustatory	79 (30.0)	85 (35.3)	0.210
Headache	88 (33.5)	89 (36.9)	0.415
Myalgia	68 (25.9)	76 (31.5)	0.159
Stomachache	23(8.7)	18 (7.5)	0.601
Diarrhea	76 (28.9)	63 (26.1)	0.489
Nausea	26 (9.9)	30 (12.4)	0.361
**Clinical conditions at admission**			
Systolic blood pressure, mean (SD), mmHg	124.6 (27.3)	123.7 (16.4)	0.650
Diastolic blood pressure, mean (SD), mmHg	73.8 (15.2)	74.8 (10.9)	0.408
Heart rate-mean (SD)	97.0 (17.594)	88.6 (15.322)	< 0.001
Respiration rate -n (%)			< 0.001
< 20	5 (1.9)	7 (2.9)	
20–29	129 (49.0)	204 (84.6)	
≥ 30	129 (49.0)	30 (12.4)	
SpO_2,_ %	86.35 (11.116)	94.3 (4.461)	< 0.001
SpO_2,_ n (%)			< 0.001
< 92%	169 (64.3)	44 (18.3)	
≥ 92%	94 (35.7)	197 (81.7)	
GCS–<15, n (%)	114 (43.3)	17 (7.1)	< 0.001
**Chest radiographs**, n = 504			
Consolidation shadow	170 (64.6)	149 (61.8)	0.513
Grand-grass opacity	239 (90.9)	209 (86.7)	0.138
Bilateral pulmonary infiltration	100 (38.0)	38 (15.8)	< 0.001
**Laboratory test**			
WBC, ×10^3^/L	6.15 (15.33)	6.99 (16.18)	0.548
Neutrophil count, ×10^9^/L	6.16 (15.36)	7.92 (17.01)	0.240
Hemoglobin, g/dL	12.6.0 (1.94)	12.92 (1.59)	0.048
Platelet, ×10^9^/L	22.29 (10.55)	26.72 (11.31)	< 0.001
Sodium, mmol/L	136.87 (6.74)	135.88 (9.38)	0.172
Potassium, mmol/L	3.76 (0.68)	3.62 (0.47)	0.006
Creatinine, mmol/L	101.72 (83.8)	76.43 (17.03)	< 0.001
Total bilirubin, umol/L, n = 441	14.37 (8.39)	12.28 (7.62)	0.017
Urea, mg/dL, n = 87	27.77 (15.90)	18.83 (15.06)	0.009
hs-CRP, mg/dL	121.31 (1286.92)	158.722 (1192.26)	0.740
Procalcitonin, ng/mL, n = 293	7.00 (18.39)	1.20 (5.08)	0.002
AST, U/mL	89.22 (292.52)	66.34 (72.44)	0.238
ALT, U/mL	136.87 (6.74)	60.90 (70.05)	0.095
D-dimer, mg/L	5.33 (11.91)	2.71 (8.31)	0.005
Serum ferritin, ng/mL	1206.68 (436.31)	906.47 (563.71)	< 0.001
IL-6 pg/ml	157.19 (434.47)	33.57 (65.98)	< 0.001
**Blood culture—**positive, (n = 68)	26 (54.2)	4 (20.0)	0.010

Abbreviation: SpO_2_, percutaneous oxygen saturation; FiO_2,_ fraction of inspired oxygen; GCS, Glasgow Coma Scale; SD, standard deviation; WBC, white blood cell; hs-CRP, high-sensitivity C-reactive protein; IL, interleukin; ALT, alanine transaminase; AST, aspartate transferase.

Symptoms present at the time of admission were not significantly different between non-survivors and survivors, except there was a higher mean body temperature (*p* < 0.001) and higher prevalence of dyspnea (*p* = 0.042) among non-survivors than survivors. Regarding patients’ clinical condition at admission, both heart rate (*p* < 0.001) and percutaneous oxygen saturation (SpO_2_) were lower (*p* < 0.001) among non-survivors than survivors, and 43.3% of non-survivors had a Glasgow Coma Scale score less than 15 at the time of admission. The results of laboratory testing showed that total bilirubin, urea, procalcitonin, D-dimer, and interleukin 6 were significantly different between non-survivors and survivors.

Although the number of patients who tested positive in bacterial culture was small (n = 68), 52.2% and 20.2% of non-survivors and survivors, respectively, had positive culture results (*p* = 0.010).

### Complications and clinical time-course in non-survivors and survivors of COVID-19

The results of a comparison of complications and the clinical time course between non-survivors and survivors of COVID-19 are shown in Table 3.

**Table 3:** Comparison of complications, clinical time course, and outcomes among survivors and non-survivors of COVID-19 infection.

	Non-survivors (n = 263)	Survivors (n = 241)	*p* value
**Complications—**n (%)			
Heart failure	6 (2.3)	5 (2.1)	0.874
Renal failure	13 (4.9)	2 (0.8)	0.007
ARDS	261 (99.2)	108 (44.8)	< 0.001
**Clinical time-course—**median (IQR), days			
Days from onset to hospital admission (n = 466)	8 (5–12)	7 (4–10.5)	0.004
Days from diagnosis to hospital admission	7 (4–11)	7 (3–10)	0.085
Days of hospitalisation	7 (4–11)	12 (9–17)	< 0.001
Days from onset to hospital discharge (n = 466)	16 (12–22)	21 (15–27)	< 0.001

Abbreviation: ARDS, acute respiratory distress syndrome; IQR, interquartile range.

In total, 99.2% of non-survivors and 44.8% of survivors developed acute respiratory distress syndrome (ARDS) during hospitalisation (*p* < 0.001). Among 369 COVID-19 patients who developed ARDS, the mortality was 70.7%.

**Fig 1:**
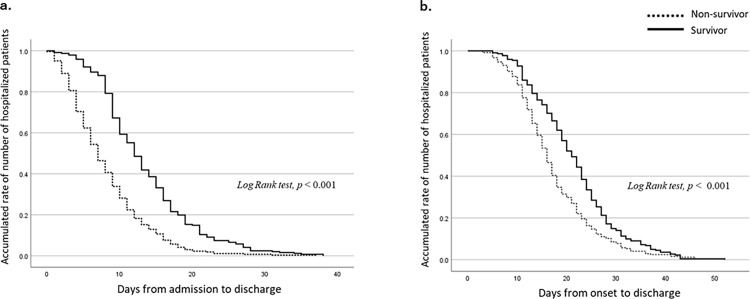
Kaplan–Meier curves showing the number of days from hospital admission to hospital discharge (a.) and the number of days from illness onset to hospital discharge (b.).

The duration (in days) from illness onset to hospital admission was significantly longer in non-survivors than in survivors (*p* = 0.004). On the other hand, the duration of hospitalisation was shorter in non-survivors than in survivors (*p* < 0.001). We assessed the number of days for hospitalisation and from onset to hospital discharge using the Kaplan–Meier method and compared non-survivors and survivors using the log-rank test (Fig 1A and 1B). The results indicated significant differences between the groups in both comparisons (*p* < 0.001).

### Treatment for patients with COVID-19

The results of comparison between non-survivors and survivors of COVID-19 regarding drug regimens and oxygen therapy are shown in Table 4.

**Table 4:** Treatment for patients with COVID-19.

	Non-survivors (n = 263)	Survivors (n = 241)	*p* value
**Drug regimens**			
Antibiotics	259 (98.5)	178 (73.9)	< 0.001
Remdesivir	136 (51.7)	194 (80.5)	< 0.001
Antifungal	20 (7.6)	4 (1.7)	0.002
Dexamethasone	213 (81.0)	208 (86.3)	0.108
Duration of treatment with dexamethasone, median (IQR), days	4 (2–7)	8 (5–10)	< 0.001
Methylprednisolone	41 (15.6)	28 (11.6)	0.195
**Oxygen therapy, mechanical ventilation**			
Oxygen mask	42 (16.0)	58 (24.1)	0.023
Oxygen Reservoir Bag	41 (15.6)	107 (44.4)	< 0.001
Nasal cannula	100 (38.0)	79 (32.8)	0.219
High-flow nasal cannula	134 (51.0)	104 (43.2)	0.080
BIPAP	11 (4.2)	10 (4.1)	0.985
Invasive mechanical ventilation	258 (98.1)	20 (8.3)	< 0.001

Abbreviation: BIPAP, biphasic positive airway pressure; IQR, interquartile range.

Among a total of 504 patients with severe COVID-19, 437 (86.75%) were treated with antibiotics and 24 (4.8%) were treated with antifungal agents. The proportion of patients treated with both agents was significantly higher in non-survivors (*p* < 0.001 for antibiotics, *p* = 0.002 for antifungals) than survivors. Remdesivir is available in Vietnam for the treatment for patients with COVID-19; the prevalence of Remdesivir administration was higher among survivors (80.5%) than non-survivors (51.7%) (*p* < 0.001). In Vietnam, dexamethasone has been commonly used in critical cases of COVID-19; this was administered in 81.0% of non-survivors and 86.3% of survivors (*p* = 0.108). However, the median duration of treatment with dexamethasone was 8 days in survivors and 4 days in non-survivors (*p* < 0.001)

As a resource-limited country, oxygen therapy is the main treatment in Vietnam for cases of severe COVID-19 infection. Oxygen was administered to all critical patients with COVID-19 in the present study. Among them, 98.1% of non-survivors were treated with invasive mechanical ventilation. Among survivors, use of an oxygen reservoir bag (44.4%) or high-flow nasal cannula (43.2%) were the main methods of oxygen therapy.

### Risk factors for mortality among patients with COVID-19 using logistic regression

The results of a logistic regression model using baseline factors indicated that diabetes mellitus, respiration rate, SpO_2_, vaccination status, time from illness onset to hospital admission, and age 60–70 years and ≥ 71 years were risk factors for mortality among patients with severe COVID-19 (Table 5).

**Table 5:** Risk factors of mortality among patients with COVID-19 using a logistic regression model.

	Coefficient	SE	P value	Odds ratio	95% CI
Diabetes mellitus	0.776	0.272	0.004	2.173	1.274–3.705
Respiration rate	-1.102	0.251	<0.001	0.332	0.203–0.543
SpO_2_	-1.710	0.249	<0.001	0.181	0.111–0.295
Vaccination status-not vaccinated	0.509	0.211	0.016	1.663	1.101–2.513
Time from onset to hospital admission	-0.041	0.021	0.050	0.960	0.921–1.000
Age 60–70 years	1.080	0.248	0.000	2.944	1.810–4.787
Age 71 and over	1.334	0.399	0.001	3.795	1.736–8.299

Abbreviation: SpO_2_, oxygen saturation; SE, standard error; CI, confidence interval.

## Discussion

In the present study, we investigated the data of patients with severe COVID-19 who were admitted to an ICU center during the outbreak of the SARS-CoV-2 Delta variant in Vietnam. Our findings revealed that the case fatality rate was high (52.2%) and most patients developed ARDS, leading to death. Delayed access to health care, poor availability of vaccines, and bacterial infections are crucial factors contributing to the mortality of COVID-19.

During the initial months of the COVID-19 pandemic, Vietnam was relatively successful in controlling the spread of COVID-19. However, since the spring of 2021, the Delta variant has contributed to a rapid rise in COVID-19 cases throughout parts of Asia. The number of daily cases in Vietnam rose gradually, followed by a sharp increase from the end of July 2021. Between January 2021 and October 2021, Vietnam had the highest number of confirmed cases per 100,000 population worldwide (12,291 cases per 100,000 population) [4], which was higher than the number in New Delhi, Bangkok, Gauteng, Jakarta, Manila, Singapore, London, New York City, and Tokyo. Just prior to the observation period of the present study, many people in Ho Chi Minh City were unable to receive medical care at the hospital, even if they had COVID-19, owing to a collapse of the health care system. The study site treated many patients with COVID-19 who had been ill at home and who had to wait to receive treatment for their illness before they admitted to the BMH ICU center, including the patients in the present study. In fact, the number of observed 504 patients with COVID-19 in the present study was all patients who were treated in the study site just in a month. Even after the BMH ICU center was set up, the median duration from illness onset to hospital admission was 7 days and 8 days in non-survivors and survivors, respectively. The BMH ICU center was able to receive the limited number of patients with COVID-19, so that the conditions of patients might be progressed to the critical conditions before they admitted to the hospital. In fact, especially for non-survivors, approximately 65% of non-survivors presented less than 92% of SpO_2_ at the time of admission. Delayed access to medical care may have caused many patients to develop critical illness before they could be admitted to a hospital, resulting in delayed initiation of necessary treatment for patients with COVID-19. The evidence to date points to an increased risk of severe illness among patients infected with the Delta variant, in comparison with other SARS-CoV-2 variants [12]. Our study results also revealed that the time from illness onset to hospital admission was a risk factor for mortality.

Another reason for the high case fatality rate among patients with COVID-19 in the ICU may be owing to secondary infection. Oxygen and steroid therapies are important treatment methods, especially in low- and middle-income countries. In our study in Vietnam, oxygen was administered for all patients and steroids was given in over 80% of patients whereas the antiviral agent (Remdesivir) was administered in approximately half of non-survivors. Broad-spectrum antimicrobial agents were administered in most non-survivors. Severe COVID-19-related pneumonia can result in an inflamed alveolar space, which provides an ideal environment for microbial growth [13]. These are all risk factors for the development of bacterial or fungal superinfection [14–16]. In addition, the study site was a temporary facility established in response to the dramatic rise in the number of patients with COVID-19 in Ho Chi Minh City. It is unknown whether this facility was constructed with sufficient infection control to prevent secondary infections. Patients with COVID-19 who had superinfection had a significantly higher mortality rate [17]. Although the number of patients with culture results was limited in the present study, approximately half of patients tested positive on blood culture. In total, 98.5% and 7.6% of non-survivors were treated with antimicrobial or antifungal agents, respectively. Additionally, patients with COVID-19 during the Delta variant epidemic in Vietnam were required to remain at home or at the local hospitals until they could be admitted to a hospital that can provide the critical care. It is possible that some patients acquired secondary infection before BMH ICU center, which led to an increase in in-hospital mortality.

In the present study, a high prevalence of patients with COVID-19 developed ARDS, especially non-survivors (99.2%). Additionally, among patients who developed ARDS, mortality was 70.7%; this was higher than that in our previous study examining 126 patients with ARDS in Vietnam prior to the COVID-19 pandemic [18]. This result is in line with the findings of a previous study reporting worse outcomes of COVID-19-induced ARDS in comparison with ARDS related to other conditions [19]. The result is also supported by studies indicating that COVID-19-induced ARDS differs from typical ARDS with a thrombotic microangiopathy [19–21], with a worse outcome in comparison with ARDS related to other conditions. A study suggested that lung protective ventilation should be implemented in all mechanically ventilated patients with COVID-19 ARDS; however, for patients with COVID-19 in the present study, the study site needed to treat over 500 of critically ill patients with COVID-19 in one month; therefore, it can be said that providing well-controlled lung protective ventilation therapy may difficult in the temporary ICU center. This may one reason for the high mortality owing to COVID-19-related ARDS in the present study. Also, although the Berlin criteria define the diagnosis of ARDS as onset within 1 week of a known clinical insult or new or worsening respiratory symptoms [22], many patients with COVID-19 in the present study may have already clinically developed ARDS before the admission of BMH ICU center; however, these patients could not be diagnosed as having ARDS owing to the limited provision of medical care during the outbreak in the referral organizations [17]. In fact, non-survivors presented with more severe conditions than in survivors at the time of admission. ARDS is a critical disease with poor prognosis, and it is crucial to control and improve symptoms such as pulmonary inflammation, thick airway mucus secretions, elevated levels of proinflammatory cytokines, lung injury, and microthrombosis in patients with ARDS owing to SARS-CoV-2 infection [23]. Greater attention is needed regarding the development of ARDS in patients with COVID-19 who are admitted in severe condition so as to provide timely treatment.

At the time of the present study, the vaccination rate was relatively low in many Asian countries. In Vietnam, although vaccination started prior to the epidemic involving the Delta variant, the proportion of vaccinated residents of Vietnam was low at the time of the Delta outbreak. In fact, among patients with severe COVID-19 in the present study, the proportion who were unvaccinated and the proportion who had received one dose of COVID-19 vaccine were higher among non-survivors than survivors in our study. The vaccination status was selected as a risk factor for mortality in a logistic regression model. Although a single dose of vaccine against the Delta variant was less effective than one-dose and two-dose vaccination against all other variants [24], the results of our study indicated that even single-dose vaccination contributed to protection against mortality among patients who were admitted in critical condition.

The limitations of the present study are associated with its retrospective design. Causality between risk factors and mortality could not be proven. The study site was urgently set up in Ho Chi Minh City in response to the epidemic owing to the Delta variant, and medical providers had great difficulty caring for the enormous number of patients with COVID-19. Because the data were collected from the medical records during a public health crisis, there were limitations in collecting all necessary data from all eligible patients, including for the assessment of ARDS severity according to the Berlin definition. As all COVID-19 patients were transferred from different hospitals in the Ho Chi Minh City area, the medical treatments that patients received prior to admission to the BMH ICU center affected their disease severity at the time of admission. We conducted the present study during the Delta variant epidemic in Vietnam; however, no included patients with COVID-19 underwent genomic analysis to confirm infection with a SARS-CoV-2 mutant virus strain. However, according to sequencing data at the time of the study period in Ho Chi Minh City, COVID-19 was almost entirely caused by one lineage of the delta variant (AY.57) [25]. In that sense, we evaluated a relatively homogeneous patient population in virologically.

Despite these limitations, the results of the present study revealed the crucial risk factors for mortality in patients with severe COVID-19 owing to the Delta variant in Vietnam, providing important information to develop a clinical strategy for patients with COVID-19 in Vietnam and other countries, especially low- and middle- income countries.

## Conclusions

During the epidemic wave involving the SARS-CoV-2 Delta variant in Vietnam, mortality among patients with severe COVID-19 in the ICU was very high. Most non-survivors developed ARDS, leading to death. It is crucial to develop systems to facilitate early access to health care and availability of COVID-19 vaccines and to prevent secondary bacterial and fungal infections, which are important factors for decreasing mortality owing to severe COVID-19, especially in low- and middle-income countries.

## Supporting information

S1 Checklist Questionnaire on inclusivity in global research. (DOCX)

S1 Dataset Deidentified original dataset. (XLSX)
